# A screening tool for predicting gatekeeping behaviour

**DOI:** 10.1002/nop2.83

**Published:** 2017-05-07

**Authors:** Austyn Snowden, Jenny Young

**Affiliations:** ^1^ Edinburgh Napier University Edinburgh EH11 4BN UK

**Keywords:** burden, concurrent analysis, gatekeeping, gate‐keeping, nurse, palliative care, paternalism, recruitment, research methods, screening tool, vulnerable populations

## Abstract

**Aim:**

To develop a typology and screening tool for gatekeeping behaviours by nurses responsible for recruitment in palliative care research.

**Design:**

Concurrent analysis.

**Method:**

Two focus groups were conducted in 2015 with nine qualified hospice community nurses involved in recruitment to a trial in palliative care. The literature was searched for research into gatekeeping from 2000–2016. All narrative examples of gatekeeping activity were coded using gerunds. Common codes were then grouped and interpreted as a social process.

**Results:**

Gatekeeping is normal and should be expected. A continuum typology emerged, ranging from unintentional to active disengagement. Justification ranged from forgetting to deliberately not mentioning the study for fear of burdening patients. Viewing gatekeeping as a continuum allowed for the creation of a screening tool designed to collaboratively discuss and hence mitigate specific types of gatekeeping behaviour before they occur. This is a unique international contribution to this persistent issue.

## 1 Introduction

Recruitment problems are common in research studies (Treweek et al., 2010). Problems are particularly acute in populations characterized as “vulnerable”, such as terminally ill patients, or patients with mental health issues (Bond Sutton et al., 2003; Witham, Beddow, & Haigh, 2013). It is suggested that 50% of randomized control trials fail to recruit to their target number (Fletcher et al., 2012). In the palliative care setting, the focus of this paper, Hanson et al. (2014) claim that 80% of studies struggle to recruit sufficient numbers.

Successful recruitment has been associated with: good communication with all stakeholders, clear protocols, “buy in” from nurses, good support from the research team, management support and the use of dedicated recruitment personnel (Caldwell et al., 2010; LeBlanc et al., 2013; McDonald et al., 2006; Treweek et al., 2010; Watson & Torgerson, 2006). Common barriers to recruitment include the reverse of these factors. However, a further common difficulty, particularly but not exclusively in palliative care research, is “gatekeeping” (Bucci et al., 2015; Sharkey et al., 2010; Witham et al., 2013). Gatekeeping in this paper refers to the prevention of access to eligible patients for research recruitment (Sharkey et al., 2010).

Kars et al. (2015) suggest there are five groups of gatekeepers:

- Healthcare Practitioners (physicians, nurses and allied healthcare workers),
- Research ethics committees (RECs),
- Management,
- Relatives,
- Researchers.

All areas are touched on in this paper. This paper focuses on gatekeeping behaviour in nurses, because they represent the largest potentially remedial cause of under‐recruitment (Stone et al., 2013).

### 1.1 Background

Gatekeeping is unethical (Sharkey et al., 2010). It conflicts with the evidence that patients want to be given the choice to participate in research (Bellamy, Gott, & Frey, 2011). However, this argument alone does not seem to be convincing enough to change gatekeeping behaviour. Consequently, a deeper, more balanced analysis of why gatekeeping persists is needed.

The literature contains thousands of reflective pieces borne of frustration at recruitment failure (e.g. Finlayson, 2015). A recent systematic review on gatekeeping activity in palliative populations alone found 1865 papers written since 2000 (Kars et al., 2016). However, primary research into gatekeeping was not the focus of any of these papers. Instead Kars et al.'s (2016) review sample had to be constructed from literature that discussed gatekeeping as part of wider investigations into attitudes to research and barriers to recruitment more generally. Therefore, despite the huge amount of commentary, there is clearly a need to conduct primary research into gatekeeping activity (Kars et al., 2016).

Kars et al. suggested that *patients* should be the focus of this research, but this begs the question of how they could be, given that gatekeepers may not permit access to relevant patients in the first place. The more logical step is to conduct primary research into *nursing* behaviours associated with gatekeeping activity in palliative care research. If these can be better and more sympathetically understood, then perhaps some of these behaviours could be mitigated where appropriate to do so.

### 1.2 Research question

What are the common actions taken by nurses in the process of preventing patients from participating in palliative care research?

## 2 The study

### 2.1 Design

Concurrent Analysis (Snowden & Martin, 2010a,b), explained in detail below.

### 2.2 Data collection

To obtain relevant primary data, two focus groups were conducted in August 2015 with a total of nine participants. All participants were qualified hospice community nurses. Age ranged from 44 to 58 years with mean 53 years. Eight were female, one was male. All were considerably experienced nurses with average time since qualification of 31 years.

Participants were asked about their experiences of identifying suitable participants for the study (Table 1) and any challenges associated with this process. JY conducted the focus groups using the semi‐structured schedule in Table 2. The structure of the focus group followed long‐standing principles originally articulated by Stewart and Shamdasani (1990), such that questions were constructed to move from the general to the specific, but remain open enough to include all contributions. Each focus group lasted between 35 and 40 min. The recordings were transcribed verbatim (Bailey, 2008) by JY.

**Table 1:** The case study

The case study
A randomized, controlled trial began in 2013 to examine the impact of holistic needs assessment (HNA) in community palliative care. Holistic needs assessment is: “…a process of gathering and discussing information with the patient and/or carer/supporter in order to develop an understanding of what the person living with and beyond cancer knows, understands and needs. This holistic assessment is focused on the whole person, their entire well‐being is discussed – physical, emotional, spiritual, mental, social, and environmental. The process culminates when the assessment results are used to inform a care plan.” (National Cancer Survivorship Initiative 2013) The UK Gold Standards Framework and NICE guidelines (2004) promote the importance of holistic assessment in palliative care, yet there is limited research on the impact of this intervention. Therefore, a protocol was developed to examine its efficacy, consistent with a comparable study in acute care (Snowden et al. 2015). Two community hospice teams volunteered to participate. The teams provided care to individuals across two contrasting geographical areas; one urban and one rural. Community nurses were required at their routine home visits to invite patients with a diagnosis of cancer to take part. Inclusion and exclusion criteria were: **Inclusion**: Community outpatient under the palliative care of the site. Over 18, capable of informed consent and expresses a wish to participate. Diagnosed with cancer. **Exclusion criteria**: Non English speaker Person deemed incapable of consenting to participate as defined by the Adults with Incapacity Act (2000)l Individuals that are in the last weeks of their life as identified by a member of the clinical care team. If patients agreed then the clinician would either integrate the HNA into their visits (experimental group) or not (control group). All consultations were to be audio‐recorded and then analysed by the research team to establish the impact of the intervention (Snowden et al., 2015).The NHS and university ethics committees had approved the study and the participating organizations were keen to be involved. The research team was multi‐disciplinary including an international team specializing in conversation analysis (Lussier et al. 2013). For sufficient power the study needed 60 participants in each arm, 120 in total. Before recruitment began the clinicians received training in holistic needs assessment from a clinical psychologist who specialised in psycho‐oncology. The research team attended six staff meetings and informal visits to offer ongoing support to the clinicians throughout the trial period. However, after 2 years only 10 participants had been recruited in total and so the trial was stopped.

**Table 2:** Semi‐structured interview schedule

Questions and prompts
Can you tell me about your experiences identifying eligible patients from your caseload? How did you decide who was suitable? How differ from inclusion/exclusion criteria already set?
How did you go about asking patients to take part? Explore difficulties. Explore confidence. Any good experiences.
Did your clinical role and the research you were asked to do complement each other or were there difficulties? Perception of research. Any other work pressures.
Do you think you wanted to protect your patients from the research? Why? How can we give these patients a voice?
What do you see as the benefits of this research? Positive benefits. Wider benefit and purpose of the research study.
How could we have provided more support Was training suitable? More input? Wider reflections.
Any further thoughts.

Relevant literature was obtained by updating the original search strategy conducted by Kars et al. (2016) in PsychInfo, Embase, Cinahl and Medline:

(gatekeep* OR gate‐keep* OR impediment OR impediments OR barrier OR barriers OR challenge OR challenges OR refusal to participate) AND (palliative care OR end of life care OR end‐of‐life care OR terminal care) AND (particip* OR respondent OR respondents OR patient OR patients) AND (research OR clinical trial OR study) NOT (dementia OR newborn)

This generated an additional 462 papers published since 2015 (the year of first acceptance of the paper). Screening for duplicates, then reading titles and abstracts for relevant primary research including nurses in palliative care reduced these to just one further empirical article, a small but important qualitative exploration of palliative care patient involvement in research (Froggatt et al., 2015).

Sections of this paper that discussed gatekeeping activity were identified alongside similar sections that included the nursing perspective from Kars et al.'s original search. Details of all papers and the sections where gatekeeping was specifically discussed are in Table 3. Quality appraisal criteria were not applicable in this study because none of the included papers were primary research into gatekeeping and so the quality of the original paper was not relevant. In other words, the purpose of the literature review was not simply to update Kars et al.'s work but to use their search terms to identify literature most likely to contain relevant sections of narrative data suitable for concurrent analysis.

**Table 3:** Kars et al.'s (2016) lit review updated and relevant data for concurrent analysis identified

Reference	Discusses nurses?	Type of article and section gatekeeping was addressed	Country	Aim of original study	Method and data collection in original study	Sample original study	Contains data suitable for Concurrent Analysis?
(Bullen et al., 2014)	Yes	Retrospective case study: discussions	AU	Identify barriers to research in palliative care	Case study	Retrospective process review	Yes
(Casarett, Karlawish, & Hirschman, 2002)	Yes	Original article: findings	US	Assess hospice staff readiness for research and attitudes to barriers to research	Survey and telephone interviews	Random sample of hospices (*n* = 79)	Yes.
(Casarett, Kassner, & Kutner, 2004)	No	Original article: findings	US	Testing of a hypothetical screening tool to select patients for research into pall care	Cross‐sectional study	Patients or carers as proxy (*n* = 214)	No
(Chen et al., 2014)	Yes	Original article: findings	US	Identify barriers to palliative care research	Qualitative Interviews	Nurse researchers (*n* = 61)	Yes
(Daniels & Exley, 2001)	Yes	Original article: findings	UK	To explore the experiences of nurses involved in recruitment of terminal patients in an RCT	Qualitative semi‐structured interviews	Specialist nurses (*n *= 10) and lead researcher (*n *= 1)	Yes
(Ewing et al., 2004)	No	Retrospective review/commentary	UK	To investigate the level of agreement on symptom assessment between patients and primary care professionals	Commentary	Retrospective review	No
(Froggatt et al., 2015)	Yes	Original article: findings	UK	To describe the experiences of people's participation in patient and public involvement (PPI) in supportive and palliative care research, specifically with respect to the benefits and challenges of participation for the individuals and the broader research support structures.	Qualitative exploratory	Patients involved in PPI (*n* = 9)	Yes
(Gardiner et al., 2010)	No	Retrospective review	UK	To explore the ethical challenges associated with pall care research	Commentary	Retrospective review	No
(Gibbins et al., 2013)	Yes	Feasibility study: discussion	UK	To establish likelihood of death during admission and test process to involve those identified in research	Observation	Patients (*n* = 327)	Yes
(Hanratty et al., 2012)	No	Comparative analysis: N/A	UK	Compare recruitment of patients and bereaved carers from general practices in areas with different research network support, and identify challenges in obtaining samples representative of those in need of end‐of‐life care	Qualitative Interviews	Patients (*n* = 13) and carers (*n* = 118)	No
(Hanson et al., 2014)	Yes	Original article: findings	US	To evaluate strategies to support recruitment in palliative care RCT	Qualitative semi‐structured interviews	All PIs and research coordinators (*n* = 18)	Yes
(Hickman, Cartwright, Nelson, & Knafl, 2012)	Yes	Original article: findings	US	Investigate strategies designed to assuage ethical concerns in palliative care studies	Case studies	PIs of 43 studies	Yes
(Hopkinson, Nm, & Macmillan, 2005)	Yes	Original article: findings	UK	Exploration of appetite for participation in research by people with advanced cancer	Qualitative	Nurses responsible for recruiting 233 patients	Yes
(Kirsh et al., 2004)	Yes	Original article: findings	US	Survey staff attitudes to research with dying patients	Survey	Hospice staff (*n* = 227) of which 67 were nurses	Yes
(Kutner et al., 2010)	Yes	Retrospective report of the researchers' experiences concerning study procedures	US	To investigate the efficacy of massage therapy for decreasing pain	RCT	Patients life expectancy of 3 weeks from 15 hospices (*n *= 380)	Yes
(Ling, Rees, & Hardy, 2000)	No	Original article: findings	UK	Investigate barriers into recruitment to clinical trials in palliative care	Survey	Patients (*n* = 558) eligible for recruitment into various trials	No
(McMillan & Weitzner, 2003)	Yes	Retrospective descriptive analysis: discussion	US	Clinical trial examining quality of life issues	RCT	Hospice patients & caregivers (*n *= 150)	Yes
(O'Mara, Germain, Ferrell, & Borneman, 2009)	Yes	Original article: findings	US	Investigate recruitment challenges in pall care research	Survey and follow up telephone interview	PIs of 15 funded research projects	Yes
(Payne, Field, Rolls, Hawker, & Kerr, 2007)	Yes	Reflective account using case studies: discussion	UK	To investigate existing palliative care provision and bereavement care (in adults and children)	Mixed methods	Wide range of participants over three studies	No
(Ross & Cornbleet, 2003)	Yes	Original article: findings	UK	Comparison of patient and staff views on participating in hypothetical research studies in palliative care.	Observation	Patients (*n* = 40) and staff nurses (*n* = 13)	Yes
(Steinhauser et al., 2006)	No	Reflection on recruitment in a longitudinal study from the researchers'	US	To construct in depth analysis of patient/carer experience of illness and death	Longitudinal study	Dyads of seriously ill patients (*n* = 240)	No
(Stevens et al., 2003)	No	Original article: N/A	UK	Investigation into ethics committees exploring the need for specialist review in pall care	Interviews	Chairs and vice chairs of UK ethics committees	No
(Stone et al., 2013)	Yes	Original article: findings	UK	Identify factors adversely affecting recruitment in pall care studies	Observational study examining eligibility, accessibility and consent.	Referrals to 18 palliative care services (*n* = 12,412)	Yes
(Tan, Wilson, Olver, & Barton, 2010)	Yes	Original article: findings	AU	To describe experience of participants in a research study into a spiritual care intervention in pall care	Qualitative: semi‐structured interviews	Hopsice staff members (*n* = 14)	Yes
(White, Gilshenan, & Hardy, 2008)	Yes	Original article: findings	AU	To determine which trial‐related factors might influence a healthcare professional's decision to refer a patient	Questionnaire	Doctors (*n* = 122) and nurses (*n* = 68)	Yes
(White, Hardy, Gilshenan, Charles, & Pinkerton, 2008)	No	Original article: N/A	AU	Logistic regression of patient willingness to participate in above trial	Questionnaire	Patients (*n* = 101) and carers (*n* = 101)	No

### 2.3 Ethics

Permission to undertake the study was granted by NHS Scotland REC 4 WS/13. All focus group participants were provided with study information, all opted to participate and signed consent forms. They were assured their contributions would be anonymized and that they could withdraw from the study at any time.

### 2.4 Data analysis

Concurrent Analysis is a method of simultaneously analysing primary data alongside relevant secondary data where the focus of enquiry is the same. It has its roots in constructivist grounded theory in that its purpose is to illuminate social processes (Charmaz, 2009). It moves away from grounded theory in relation to the role of the literature. Grounded theorists have traditionally needed to make a decision as to whether to engage with relevant literature before or after gathering primary data (Dunne, 2011). Concurrent Analysis is grounded in a rejection of this debate altogether (Snowden & Atkinson, 2012). It takes an alternative position by analysing the primary data at the same time as relevant elements of the literature.

The method has successfully been used in previous studies designed to better understand satisfaction in childbirth (Hollins‐Martin, Snowden, & Martin, 2012; Snowden et al., 2011), the process of becoming competent as a nurse prescriber (Snowden, 2010; Snowden & Martin, 2010a,b) and the process of organizational change associated with implementing an electronic health record (Snowden & Kolb, 2017). It is a pragmatic method of synthesizing primary data with literature to produce more generalizable results than doing either alone (Snowden & Martin, 2010a,b). For a detailed description of the philosophy of Concurrent Analysis please see Snowden and Atkinson (2012).

The process of Concurrent Analysis involves four stages:

1. The gathering, transcription and collation of all relevant data.Relevant data include all narrative research data focused on the topic of interest. In this study, it meant primary data gathered in the focus groups obtained to answer the research question. It also included the relevant sections of the papers identified in Table 3, where either researchers or participants in their study described gatekeeping behaviour. All data were imported into NVivo 10 for coding.
2. Line by line coding of the data focusing on gerunds.The purpose of Concurrent Analysis is to identify a social process. Concurrent Analysis considers the units of social process to consist of *actions* taken by participants (Snowden & Atkinson, 2012). Coding at this level, therefore, looks for words describing action or behaviour such as gerunds for example (Charmaz, 2006). For example, “forgetting” is a gerund, as is “being too busy”.
3. Identification of commonalities and connections between codes.During the line‐by‐line coding, commonalities are flagged for further analysis. For example, if a significant proportion of participants mention that they imagine that partaking in research would be burdening their patients, then “burdening” is identified at this stage as a potentially important category. Connection is maintained at this point as to who is discussing “burdening” and under what circumstances. This maintains the context for the codes thus providing explanation for a given behaviour. For example, “burdening” may mainly occur in conjunction with participants describing the physical or mental state of the potential participant. These are important connections to maintain.
4. Thematic grouping of key themes to explain the whole as a social process. The final phase is to examine all the codes, connections and themes in relation to each other. Where actions are widely reported these are considered key themes. All the key themes are then considered as a whole to see if an underlying social process can be described to explain them all.

### 2.5 Rigour

Both authors independently coded all the data and then came together to discuss anomalies in interpretation. These were rare and were resolved by discussing and then agreeing on the most coherent rationale for differing judgements. Both authors were involved in all stages of the analysis. Agreement was reached on the inclusion of all themes and the data summaries discussed next. A version of the interpretation and subsequent screening tool was presented to an audience of over 50 nurses at the RCN International Research Conference in Edinburgh (Snowden & Young, 2016). Formal feedback was not gathered, but the debate that followed the presentation suggested that the findings were generalizable to that particular audience, adding a final external element of rigour (Morse, 2015).

## 3 Results

The key interpretation was that there is a continuum of gatekeeping activity. The continuum from nurses forgetting about the research study (unconscious aspects) to actively disengaging from it (conscious aspects). These actions are a function of a range of causes from simple distractions through to discomfort and distress. Nurses give a generalizable range of reasons for their behaviour, including seeing research as a burden for both themselves and the patient, or seeing research as a low priority in relation to more pressing clinical issues. Regardless of cause or explanation, the outcome is the same: the patient is not asked to participate in the study.

**Figure 1:**
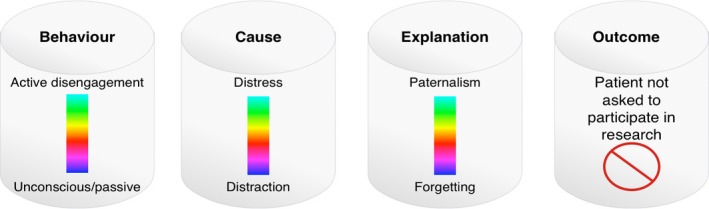
A typology of gatekeeping activity including its cause and outcome

This typology is summarized in Fig. 1. The key themes and subthemes are presented alongside supporting evidence from the literature and focus groups in Table 4.

**Table 4:** Themes, their causes and explanation from the focus group and the literature

Theme/Behaviour	Cause	Explanation	Examples from the literature	Examples from the focus groups
Active disengagement	Distress	Incongruent with development of therapeutic relationship	I'm not in favour of clinical staff doing research… often, patients have very short lengths of stay which prohibits development of rapport.(Kirsh et al. 2004, p. 277)	It takes a few visits to build up trust and a relationship with the patients and I think jumping in with a study initially is wrong. I think that could be detrimental to our service as patients could say “I don't want these nurses back as they're asking about studies”.
		Burden (for staff and patients)	…offering a clinical study to this population may be perceived as placing undue burden on the patient (O'Mara et al., 2009, p. 4) …a form of “catch‐22” occurs in which staff are interested in research and want to control access to it but won't get involved and actually let it happen because they do not have time to absorb the extra burden it would create. (Kirsh et al. 2004, p. 278)	I felt perhaps myself it was a huge burden to ask patients, perhaps I held off for that reason without asking them.
		A priori assumptions	“I can't imagine anyone wanting to talk about these things.” (Tan et al. 2010, p. 163)	I think I was probably making the decision for them because I thought there's no way you could take this on.
		Paternalism	“He and his wife are very, very anxious and I think would be made worse by extra ‘fuss and attention’. I therefore do not think it would be appropriate to approach them.” (Ewing et al., 2004; p. 456)	I know we often get labelled as being very parental about our patients but there is something in that because we are advocates for our patient. There are so many other things that we need to do for them, their agenda and this was our agenda so you probably do a bit of blocking.
	Discomfort	Complexity	…as trials became more complex with potential side effects, less were willing to refer.(White & Hardy, 2009; p1399).	I think it's… I've got these papers and this to fill out and I've got to read that and now I've got to use this tape and you don't have a table and a contained room and you have to go with where the patient is going, it's not all clean and tidy.
		Creating bespoke inclusion criteria	We'd be kind of thinking about a family that has a degree of cohesiveness and may have things that are unsaid that if put in this forum would be able to be brought out…[By contrast] there were some cases that were probably far too complicated with too much history and friction.” (Tan et al. 2010, p. 162)	They are very few and far between where the patient is so easy. What I think in that particular situation though, she wasn't perhaps a typical patient like I generally have on my caseload. I'm wondering what it was about her that maybe made her less typical and maybe she was generally quite well.
		Protection	The poor performance status, fatigue and low mood of many patients should not be underestimated and may make apparently straightforward procedures such as record or diary keeping too onerous for the potential trial participant. (Ross et al. 2015, p. 15)	I think we looked for a patient that would fit a certain criteria. I suppose we are all doing the same job but we are all different people doing it differently and therefore I think our selection is probably different.
	Discord	Lack of collaboration	Negative attitudes towards research held by dominant individuals within the team were evident, influencing less‐decided team members about the value of the project… it was observed that if one individual proposed a particular view there was a tendency for others to agree or remain silent rather than expressing divergent views. (Bullen et al., 2014, p. 80)	[The study protocol] was almost completed when it arrived.
Passive disengagement	Distraction	Not proper work	Research activities are not “proper work” (Woodward et al., 2007; p. 234)	To be honest I was too busy with other work…
		Not enough time	More work needs to be done to free up time of gatekeepers for involvement with research and more education needs to be provided by study teams to gatekeepers to generate interest.(Borschmann et al., 2014, p. 5)	
		Not core business	Conducting clinical research to inform nursing practice is not viewed as “core business” as it is seen to detract from a patient care focus. (Bullen et al., 2014, p. 79)	We have had staff changes, staff sickness all of that has impacted definitely, so the study got lost…I must admit I was too busy… I didn't even think about the study.

### 3.1 Active disengagement

#### 3.1.1 Distress

Several participants expressed conscious decisions to disengage from the research process. There were two main justifications for this. First was the view that research was incongruent with the development of a therapeutic relationship and therefore damaging to the development of rapport. Secondly and more frequently expressed was the belief that inviting patients to participate would be a burden for them.

Like Kars et al. (2016), Witham et al. (2013) found the explanation for considering research a burden for the patient to be grounded in particular disease‐specific discourses. Individual justifications for not “burdening” patients with details of potential participation in research were often framed in disease‐specific notions of vulnerability. For example, people with dementia or learning disability “would not understand”, while people with terminal illness “should not be bothered”. “Ill health” was given as the major rationale for gatekeeping in Hanratty et al.'s (2012) investigation.

Nurses knew they were removing choice by taking this position, but often explained their actions as morally justifiable because they were protecting the vulnerable. For example, while acknowledging the paternalistic nature of gatekeeping behaviour, the term “advocate” was used by one nurse, effectively reframing paternalism as a moral good. There was also the claim that introducing research moves away from a focus on the patient's needs. This “a priori” stance that the patient needs protecting from research is widely evident in the literature and not confined to nurses (Ammari, Hendriksen, & Rydahl‐Hansen, 2015). Gysels et al. (2008), for example, found carers asked recruiters not to approach their partners for interview as they thought the experience may harm them. Payne (2013) pointed out that patients often act to protect their carers from the “burden” of research. Protection of a loved one is an entirely understandable, rational response, as is the protection of the therapeutic relationship. It may, therefore, be helpful as a starting point to view gatekeeping as a *normal* response rather than a misguided one. People protect both themselves and others from perceived stress wherever possible (Festinger, 1957).

#### 3.1.2 Discomfort

Avoiding discomfort could explain gatekeeping behaviour that may be a function of knowledge deficits. For example, it has been shown that gatekeeping increases consistently with the complexity of the study design (White & Hardy, 2009).

Dunleavy et al. (2011) showed that nurses who do not understand randomization are unlikely to be able to explain it adequately to patients and thus consciously or unconsciously do not discuss it at all. One of the focus group participants described a failed attempt to recruit as not being “clean and tidy” (Table 4). This is quite clearly an expression of discomfort with the unfamiliar.

Extending from this were claims that those who were deemed to be suitable for inclusion were classed as being *unusual* in some way. There is evidence that nurses do not necessarily follow set inclusion/exclusion criteria (Tan et al., 2010), but rather select potential recruits on characteristics that they think may be useful to the research (Table 4).

Alternatively, Ross et al. (2015) suggest researchers may overestimate the capacity of some patients. This shows that in these instances nurses and researchers have different perspectives on what constitutes a suitable patient for any given study. The impact on the research is biased recruitment and hence invalid results. As in much of the literature (Kars et al., 2016) our study (Table 1) did not recruit to power and it is likely that those that were recruited could not be described as having consistently fit the inclusion/exclusion criteria.

#### 3.1.3 Disharmony

Team dynamics play a substantial role in the success of any initiative (Snowden & Kolb, 2017). Initially the research team had hoped that if one or two members of the nursing team reported positive experiences of recruitment then it might encourage others to engage. Unfortunately, the reverse appeared to happen. It has been recognized previously that if one or two stronger members of staff disengage it becomes much harder for others (Kars et al., 2016). A factor in this particular study may have been the fact that the study protocol had been largely developed without the involvement of the staff responsible for recruitment (Table 4). Greater collaboration between the research and clinical team during the planning stage of this project in particular could possibly have helped engagement.

### 3.2 Passive disengagement

#### 3.2.1 Distraction

The final category contains all the less conscious elements of recruitment failure. The work pattern into which the study fit was a significant factor. Where other work pressures emerged, the research became less important. Lack of time to do anything other than routine but urgent work was mentioned almost unanimously (Table 4). Research is often not considered “core business” (Bullen et al., 2014, p. 79), or even “proper work” (Woodward, Webb, & Prowse, 2007, p. 234). Some participants simply forgot about the study and this was usually explained as a function of being very busy.

## 4 Discussion

The challenges associated with recruitment in palliative care are commonly reported in the literature. Gatekeeping by nurses is known to present a substantial problem, but solutions are less clear. There is a tendency for authors of gatekeeping critiques to propose “culture change” as solution (Bucci et al., 2015; Witham et al., 2013). While this may be a desirable endpoint, it is difficult to enact and so even where the critiques are credible they demonstrate a lack of practicality. What this study adds is a categorization of types of gatekeeping activity exhibited by nurses. The function of this categorization is to help future researchers mitigate these behaviours wherever feasible and appropriate.

In summary, a range of gatekeeping responses was found, from benign unconscious forms of disengagement through to conscious and deliberate decision‐making forms. This is important because different actions may be useful to mitigate gatekeeping consistent with the particular type of gatekeeping behaviour. For example, a benign form of gatekeeping is simply forgetting to ask people (Jessiman, 2013). If this was generalizable, as seems the case here, then it could easily be addressed using various prompting techniques. A step up from forgetting included nurses justifying omission in relation to more pressing clinical priorities (Potter, Dale, & Caramlau, 2009; Kars et al., 2015). In these cases, the research was seen as separate and less important than clinical work. Where this is the case then a discussion on whether this is an accurate appraisal may be helpful. Likewise, in the cases where assumptions about burden result in gatekeeping these assumptions could be questioned, especially given that Graffy et al. (2009) concluded that the positive attitude to research of clinicians was the most important component of successful recruitment.

It is easy from an outsider perspective to argue that gatekeeping is irrational. For example, take the claim that introducing a research study would somehow breach the therapeutic relationship. The components of a successful therapeutic relationship are widely agreed. They include trust and commitment, empathy, unconditional positive regard, genuineness, honesty and support (Ramjan, 2004). It is difficult to see how asking a person to become involved in research may breach any of these, especially as there is considerable evidence that people want to participate in research for altruistic reasons (Newington & Metcalfe, 2014).

Nevertheless, the perception that damage could occur is real. This perception is shared by other health professionals, patients and their carers. Participants in our study reported genuine concern for the patient, anxiety about research competence and competing workload issues. These findings were consistent with the wider literature, suggesting that nurses show a consistent pattern of behaviour and use a consistent range of explanations to explain their behaviour.

As a consequence a more measured response to gatekeeping is needed beginning with the expectation that gatekeeping will occur (Ewing et al., 2004). On reflection, neither the researchers nor nurses understood the magnitude and type of gatekeeping behaviour that would be likely, or the reasons for it, both before and during the study. Therefore, discussing the likelihood of gatekeeping before the study has been fully conceptualized would be the best method of mitigating it where appropriate.

This discussion needs to be evidence based. To facilitate this, the typology developed here was used to construct a screening tool. The screening tool is in Table 5. It turns the key findings from the typology into statements that the responding nurse can agree with, disagree with or be undecided about. Gatekeeping likelihood and support can then be clarified. To be clear, all the nurse recruiters in the study (Table 1) were very keen to take part and had not expressed any qualms to the research team about their capacity to recruit the study sample needed. In fact, they were confident and enthused about the study. Completion of the screening tool may have facilitated a more focused, realistic discussion starting from the perspective that gatekeeping is *likely* to occur.

**Table 5:** Screening tool for likely gatekeeping activity

Gatekeeping screening tool			
Please answer every question as honestly as possible by placing a tick in the relevant box. There are no right or wrong answers and your responses will only be used for discussion purposes.			
	*Yes*	*No*	*I don't know*
I will mention research participation to every patient who meets the inclusion criteria.			
I think patients with capacity to consent should always be given the choice to make their own decisions.			
I will not mention research participation to a patient if they look as if they couldn't manage it.			
I think that research is as important as clinical work.			
Research informs my clinical practice.			
I know what I need to do to fulfill my role in this study.			
I can answer any questions the patient may have about the research.			
I sometimes forget to ask people if they want to participate in research.			

It is interesting to note that proposals designed to minimize gatekeeping are similar regardless of the subject. For example, in relation to conducting anthropological studies in indigenous populations, Kawulich (2015) recommends the following:

Establishing trusting, long‐term relationships through social networking, acquiring specific permissions at various levels, selecting key informants, presenting oneself appropriately and showing respect for cultural mores are essential aspects of being granted entry by gatekeepers.(p57)

These are transferable principles and it is widely agreed that one of the best ways of operationalizing this agenda is through deployment of dedicated recruitment personnel (LeBlanc et al., 2013). Not every study can afford such a resource, but “clinical champions” are also effective (Hanson et al., 2014). The screening tool could potentially identify those more likely to be effective in this role. Recall the introduction stated that successful recruitment is also consistently associated with good support from clinical management (Caldwell et al., 2010; LeBlanc et al., 2013; McDonald et al., 2006; Treweek et al., 2010; Watson & Torgerson, 2006). Again the screening tool could provide a snapshot of the depth of this support.

It is very important that any discussion of gatekeeping starts from a balanced and sympathetic view of why gatekeeping is likely to occur. For example, a complex and sensitive issue is the belief that participation in a research study will cause unnecessary burden to the patient. Telling nurses this may not be true is a very ineffective strategy. Discussing beliefs around advocacy, paternalism, choice and risk in a supportive collaborative environment, beginning by acknowledging the rationality of taking a protectionist stance is much more likely to be effective.

There are also practical elements of ongoing support likely to be necessary (Borschmann et al., 2014). It was clear in the focus groups that the nurses were uncomfortable with the study design and the literature is very clear in showing a relationship between study complexity and recruitment success. However, the nurses' discomfort did not come to light until after the study had been stopped. Had the screening tool been completed beforehand perhaps a more complete picture of nurse discomfort with the research methods may have emerged.

### 4.1 Limitations

It is not the aim of qualitative research to make broad generalizations. Concurrent Analysis is designed to support greater generalizability (Snowden & Martin, 2010a,b) but it could still be argued that the small sample size may not be representative of gatekeeping responses from nurses in the wider community palliative setting. Furthermore, the participants in this study were known to the researcher who carried out the focus groups. This may have hindered discussion around the challenges associated with recruitment, particularly in relation to active disengagement or frustrations at the research process. The study relied on an updated literature‐searching algorithm previously used successfully by Kars et al. (2016). While their focus was on gatekeeping and their systematic review was published in a high‐impact journal, there is the possibility that some research may have been omitted in error. Finally, this was a retrospective account, with recall going back 2 years in some cases. Prospective studies of this typology are therefore needed.

## 5 Conclusion

Despite its small primary data set, this study has original implications for future studies in palliative care. Qualitative explorations of gatekeeping activity by nurses are under‐reported, as gatekeeping activity itself is rarely the focus of the research. Therefore, despite numerous statements that gatekeeping is problematic there is a lack of understanding around how to challenge it, when appropriate. This study has developed a continuum typology of gatekeeping behaviour and gone on to construct a screening tool designed to ascertain and discuss the likelihood of these behaviours and attitudes which have an impact on recruitment. Several evidence‐based initiatives may then be used to mitigate these specific behaviours where appropriate.

The first step is to bring gatekeeping into the open at the earliest opportunity by asking recruiting nurses to complete the screening tool. This means investing time before the study begins to discuss issues such as selection criteria, perceived burden and issues of patient autonomy. Ideally, recruiting nurses should be involved throughout the research process from study funding application and design onwards and thus drive these discussions. However, studies are often constructed by external partners and then brought into practice once funded. In these cases, the potential for gatekeeping should be discussed in a collaborative and supportive manner from the start, beginning with the expectation that gatekeeping *will* occur and that this is usual. This will allow nurses to reflect on their likely responses in a safe environment and consider what they are actually likely to do when faced with typical scenarios.

If this first step is achieved the study is more likely to remain as a significant priority amongst other competing priorities. Furthermore, once a good relationship has been built between the researchers and nurses there is more likelihood that complex issues such as the patient right to participate will be broached in a critical and collegiate manner. It is a fundamental right of any patient to refuse to participate in any research, but it is also a fundamental right to be asked in the first place unless capacity is legally impaired. This decision is much more likely to be facilitated in an atmosphere of collaboration and the screening tool developed here has been designed to support this process.

## Conflict of interest

No conflict of interest has been declared by the authors.
